# Development of mental health first aid guidelines for deliberate non-suicidal self-injury: A Delphi study

**DOI:** 10.1186/1471-244X-8-62

**Published:** 2008-07-23

**Authors:** Claire M Kelly, Anthony F Jorm, Betty A Kitchener, Robyn L Langlands

**Affiliations:** 1 ORYGEN Research Centre, University of Melbourne, Australia

## Abstract

**Background:**

It is estimated that around 4% of the population engages, or has engaged, in deliberate non-suicidal self-injury. In clinical samples, the figures rise as high as 21%. There is also evidence to suggest that these figures may be increasing. A family member or friend may suspect that a person is injuring themselves, but very few people know how to respond if this is the case. Simple first aid guidelines may help members of the public assist people to seek and receive the professional help they require to overcome self-injury.

**Methods:**

This research was conducted using the Delphi methodology, a method of reaching consensus in a panel of experts. Experts recruited to the panels included 26 professionals, 16 people who had engaged in self-injurious behaviour in the past and 3 carers of people who had engaged in self-injurious behaviour in the past. Statements about providing first aid to a person engaged in self-injurious behaviour were sought from the medical and lay literature, but little was found. Panel members were asked to respond to general questions about first aid for NSSI in a variety of domains and statements were extracted from their responses. The guidelines were written using the items most consistently endorsed by the consumer and professional panels.

**Results:**

Of 79 statements rated by the panels, 18 were accepted. These statements were used to develop the guidelines appended to this paper.

**Conclusion:**

There are a number of actions which are considered to be useful for members of the public when they encounter someone who is engaging in deliberate, non-suicidal self-injury. These guidelines will be useful in revising curricula for mental health first aid and NSSI first aid training programs. They can also be used by members of the public who want immediate information about how to assist a person who is engaging in such behaviour.

## Background

A Mental Health First Aid training program was developed by Kitchener and Jorm [1] to train members of the public to assist others in getting appropriate professional help for mental disorders or assist in mental health crisis situations. The role of the giver of mental health first aid is "to assist the person until appropriate professional help is received or the crisis resolves [2]." This can involve a member of the public recognising the signs of developing mental illness and responding effectively, encouraging the person to seek professional help where needed and supporting them in the use of self help strategies when these are appropriate.

When the program was first in development, the authors used evidence-based information wherever possible, but very little research was found about how members of the public, with no clinical training, could assist a friend, family member or acquaintance who was showing signs of mental illness or crisis. This paper describes one of a series of projects to more formally develop first aid guidelines, using professional, carer, and consumer expertise. The original program did not include any discussion of NSSI or NSSI first aid. It was decided that because of increasing public awareness of NSSI, and a new course being developed for adults to offer mental health first aid to young people, it was going to be important to develop first aid guidelines for NSSI.

### Definition of self-injury

There are many different terms used to describe self-harm, including *deliberate self-harm *(DSH), *deliberate self-injury *or *self-injury *(DSI or NSSI), *self-mutilating behaviour *(SMB), and more colloquial terms such as *cutting*. In addition, the term *self-harm *is often used to describe non-fatal suicidal behaviour and suicide attempts, as well as injury inflicted with different intentions. It has even been used to describe purging behaviours in eating disorders [3].

Until recently, in the scientific literature, distinctions between self-harm which is intended to be fatal and self-harm which serves other purposes have not been delineated consistently. However, this is improving. There is a good deal of evidence available now to suggest that deliberate, non-suicidal self-injury can be distinguished from self-harm which is intended to result in death [3-15]. Many people who have made suicide attempts in the past and have also engaged in other forms of NSSI describe the two behaviours as separate from one another [15].

In the past two years many researchers have been using more specific terms including our preferred term, *non-suicidal self-injury *(NSSI) [4,5,15-19], including authors contributing to a recent special issue of the Journal of Consulting and Clinical Psychology [6-9,20-24]. Other authors have used the less preferred term *non-suicidal self-harm *(NSSH) [13,25]. For the purposes of this project, we accept that deliberate, non-suicidal self-injury is different to a suicide attempt, and from here on, we use the term 'non-suicidal self-injury' (NSSI) to describe this behaviour. We do not use the term *self-harm *because it encompasses so much and is not clearly defined.

Three major types of NSSI have been described in the literature. Stereotypic self-injury, usually observed in people with severe intellectual impairment or brain injury, delineates repetitive injuries such as repeatedly hitting one's own head with a hand or against a wall. Major self-injury, usually observed in people in a psychotic state, is a one-off dramatic act with major consequences such as self-enucleation or self-castration. Only repetitive self-injury, usually seen in people with mood disorders and personality disorders [14,15,26,27], is addressed in these guidelines, and the typical patterns and motivations are described below.

### Types of and motivations for self-injury (NSSI)

NSSI includes a wide range of behaviours. The most common forms of NSSI are pinching and scratching the skin, punching or hitting objects until marking or bleeding occurs, or cutting the skin [4,20]. Cutting may also include carving words or symbols into the skin. Other forms of NSSI include burning the skin, interfering with the healing of wounds, inserting or rubbing foreign objects into the skin, or pulling hair out by the roots (trichotillomania). More rarely, NSSI can include breaking or attempting to break bones. Self-poisoning is sometimes described as NSSI behaviour, but is more commonly associated with suicide attempt. The Deliberate Self-Harm Inventory [28,29] specifically excludes measures of self-poisoning.

The motivations for NSSI are numerous. Common motivations include: to escape from unbearable distress or anguish, to gain relief from tension, to escape a dissociative state, to express a need for help, or to change the behaviour and emotional states of others [30]. Some people say that the emotional pain they are feeling is so intense that they need to balance it with a feeling of physical pain, or that they feel nothing at all and need to injure themselves to feel something, or to observe blood and be reassured that they are indeed 'real' or 'human' [11,12,19,27-32].

It is estimated that around 4% of the population engages or has engaged in NSSI [33], and this may be increasing [30,32,34]. However, estimates vary a great deal, due in large part to the varying definitions used in epidemiological research. It is more prevalent in certain groups. A recent review reported lifetime rates of NSSI in children and adolescents ranging from 13–23%, with one year estimates of 3–13% [35]. However, a US study released after this review was published, found that 47% of a high school population (mean age 15.5 years) had engaged in some form of NSSI in the previous year and 28% had engaged in moderate to severe NSSI in this period [29]. A recent finding in college students in the US show that 17% of this group had engaged in at least one episode of NSSI in their lifetime [30].

Rates of NSSI in clinical samples have been reported at between 7–21% [33], and in psychiatric inpatient samples, as high as 60% [36]. One older study of children aged 5 to 12 years old who had experienced physical and sexual abuse found that 41% of this group engaged in NSSI, compared to 17% of a neglected group and 7% of a normal control group [37].

### Aim

The present study uses the Delphi methodology to develop first aid guidelines for NSSI. The Delphi methodology has been used in health research in the past, mainly to reach consensus amongst medical practitioners, but also with consumers of health services in some settings [38,39]. We have previously used the Delphi method to develop companion first aid guidelines for suicidal thoughts and behaviours [40], as well as mental health first aid guidelines for depression and psychosis [41,42]. No research using the Delphi methodology to determine consensus on NSSI first aid guidelines has been conducted previously.

## Methods

This study had two phases: a literature search and questionnaire development, and the Delphi process.

### Literature search

The aim of the literature search was to find statements which instruct the reader on how to offer assistance to someone who is engaging in NSSI in the short term, and how to access appropriate professional help for them. The literature search was conducted across two domains: the medical and research literature, and lay literature. The lay literature included books written for the general public, particularly carers' guides, websites and pamphlets.

The medical and research literature search was accessed through searches of PsycInfo [43] and PubMed [44]. The search terms used were 'self-injury', 'self-harm', 'self-mutilation' and 'self-inflicted'. All records for the 20 years leading to the search date were reviewed. The search terms generated too many records, in particular because the term self-harm is so frequently used in reference to suicide attempts, but all attempts to narrow the search were found to be unsatisfactory. Papers which described assessing patients for signs of NSSI, brief interventions, or guidelines for treating NSSI patients were reviewed, but there were very few of these. Most of the articles which focussed on NSSI as a set of behaviours distinct from suicidal gestures and suicide attempts were general interest articles describing what NSSI is, underlying motivations, or the high rates of NSSI in patients with personality disorders.

To find appropriate websites, we searched Google [45], Altavista [46], and Yahoo [47] using the same search terms as in the medical literature, but we also included the term 'cutting' as this is a colloquial term often used by consumers. The first 50 websites listed by each were reviewed. Beyond 50 websites, quality declined rapidly. Since most websites were listed by more than one search engine, only 79 websites were reviewed. The websites were read thoroughly, once again looking for statements which suggested a potential first aid action (what the first aider should do) or relevant awareness statement (what the first aider should know). Any external links to other websites were followed and the same process applied to each of them. As in the medical literature, we found that there was very little advice offered, beyond general advice to stay calm and be understanding.

The fifty most popular books on the Amazon [48] website which listed the words 'self-injury', 'self-harm', 'self-mutilation' or 'self-inflicted' in the title or keywords were selected. This site was chosen because of its extensive coverage of books in and out of print, including works about mental health aimed at the public. Books which were fictional, autobiographical in nature, clinical texts, or manualised self-help guides were excluded. The remaining books, mostly carers' guides, were read to find statements which suggested first aid actions. There were few relevant titles. However, other carers' guides known to the researchers, which focussed on other mental illnesses or mental illness more generally, were also examined for relevant first aid advice. Once again, very little potential advice was found. Any relevant pamphlets were sought and read, and statements were taken from these as well. The majority of the pamphlets were written and distributed by organisations focussing on mental health in general, but a small number of pamphlets have been produced by organisations with a focus on NSSI.

Across all the domains, there was only a very small amount of advice which could be adapted into first aid. Where such advice was found, it was always a variation on one of the following: be understanding, do not assume the person is suicidal, and do not act disgusted.

### Questionnaire development

Because so little advice was found across the two domains of the literature search, the first round of the questionnaire was different to the Delphi process we have used in previous studies [40-42]. In the first round, participants were asked to answer a number of open-ended questions. The questions explored aspects of NSSI which had been identified in the literature. The questions were:

1. How should a first aider enquire about scars or injuries they have noticed on someone, if they believe that they may be self-inflicted? Is it advisable to ask about such injuries, or might it do further damage?

2. If a first aider has discovered someone in the act of self-injury, how should they react, and what should they say and do?

3. Should all people who self harm receive medical and psychiatric attention?

4. Do you think that the 'harm minimisation' approach is appropriate for self injury?

5. Do you think people who frequently injure themselves should carry a first aid kit with them to reduce the risk of secondary harm (from infection, etc)?

6. Do you think that carers of people who frequently injure themselves should facilitate or restrict access to 'clean' implements at home?

Participants were encouraged to write as much as they wished to. The questions had prompts to encourage detailed answers; for example, question 3 continued "what kind of help is the most important? Does this vary between cases?"

The answers to these questions were read and analysed for actions which could be expressed as first aid recommendations. Statements were grouped into the following six categories: making the decision to intervene, what to do if you have interrupted someone in the act of injuring themselves, discussing NSSI, alternatives to NSSI, harm minimisation and seeking professional help. Similar and near-identical statements were frequently found in multiple responses, and they were not repeated in the questionnaire. A working group comprised of the authors of this paper and colleagues working on similar projects convened at each stage of the process to discuss each item in the questionnaire. The role of the working group was to ensure that the questionnaire did not include ambiguity, repetition, items containing more than one idea or other problems which might impede comprehension. The wording was carefully designed to be as clear, unambiguous and action-oriented as possible. For example, 'the first aider should find out if the injury is bad or not' is better stated, 'the first aider should ask the person if they are in need of medical assistance'. All statements were written as an instruction as shown in the above example. Items which were not included in the questionnaire were those which were so ambiguous that the working party was not able to agree on the meaning of the statement, and those which called upon 'intuition' or 'common sense', as these cannot be taught. Items which were more appropriate to clinical therapeutic practice were also excluded. An example of this is a suggestion made by a number of panel members to 'find out what function the self-injury has for the person, and find a way to accomplish that function through non-harmful actions; for example, if the person is feeling numb and wants to feel something physical, they could put their hands into ice water instead of cutting themselves'. This may be a useful thing to do, and may be a skill which many consumers and carers could learn, but it is beyond the role of a first aid giver, and is more appropriate as therapy.

All participants answered the questionnaire via the Internet, using an online survey website [50]. Participants were able to stop filling in their questionnaires at any time and log back in to continue, without losing the completed section of their questionnaires. Using the Internet also made it very easy for the researchers to identify those who were late in completing questionnaires and send reminders, with no need to send extra copies of the questionnaire. No questions were inadvertently missed, as the web survey was set up so that each question was mandatory.

### The Delphi process

Participants were recruited into one of three panels: professionals (clinicians and researchers), consumers (people who had a history of NSSI) and carers. The professional panel had 26 experts, the consumer panel 16, and the carer panel 3. All panel members were from developed English speaking countries (Australia, Canada, England, New Zealand and the United States). Participants were recruited in a number of ways. Professionals recruited were those who had publications in the areas of intervention, prevention, and/or treatment of patients who engage in NSSI. When letters were sent to professionals asking them to be involved, they were also invited to nominate any colleagues who they felt would be appropriate panel members. Those active in clinical practice were also asked to consider any former patients who might be willing to be involved. Consumers were recruited from advocacy organisations, and referral by clinicians. They were also identified if they had written websites offering support and information to other consumers, or if they had published memoirs. Carers were recruited through carers' organisations or referred by consumers on the panel. Carers were difficult to recruit for this study and, after the first round of the questionnaire, only one carer remained involved. The decision was made by the working group to keep the suggestions made by carers in round 1, but to exclude their answers to subsequent rounds.

Demographic information about the three groups is available in Table 1, which shows the age and gender of the three groups. Of the 26 professional participants, 18 were clinicians (9 clinical psychologists, 5 psychiatrists, 2 psychiatric nurses, 1 general psychologist, and 1 general psychologist who was also an art therapist). The remaining professionals included 3 CEOs of mental health organisations (one of whom was also a psychiatric nurse, counted above), 2 university lecturers and researchers, a university based researcher, a research director in a youth-focussed organisation, a consumer consultant for a professional College of Psychiatrists and a youth development worker and trainer. In addition to these professional roles, the 26 professional participants included 19 who had been published: 14 who had published academic papers and books chapters, and 9 authors of major works including self-help books, textbooks, major reference works and, in 2 cases, major websites.

**Table 1:** Study participants – age and gender

	Male	Female	18–29	30–39	40–49	50–59	60+
Consumers	2	14	7	4	4	1	-
Carers	-	3	-	1	2	-	-
Professionals	10	16	1	7	7	8	3

Of the 16 consumer participants, all had taken higher-level roles in consumer advocacy (sometimes multiple roles), for example, training, liaison roles and support group facilitation. 5 had produced major works of writing such as books and 9 had contributed to academic papers and articles in consumer-oriented publications. The three carers had roles in mental health organisations, and additional roles such as authoring academic papers, offering training and mental health nursing.

After the initial questionnaire, which solicited suggestions for first aid actions, three rounds of questionnaires followed. Each statement in the questionnaires was rated up to two times. In round 1, the questionnaire derived from the process described above was given to the panel members. The questionnaire included space after each of the sections to add any suggestions for new statements that panel members felt should be included.

In each round of the study, the usefulness of each statement for inclusion in the mental health first aid guidelines was rated as *essential*, *important*, *don't know *or *depends*, *unimportant*, or *should not be included*. The options *don't know *and *depends *were collapsed into one point on the scale because operationally, they are the same response. Most of the statements were, very reasonably, noted to be useful in some cases and not others, meaning they could not be generalised into guidelines, which was also true of statements participants did not feel confident to rate.

**Figure 1:**
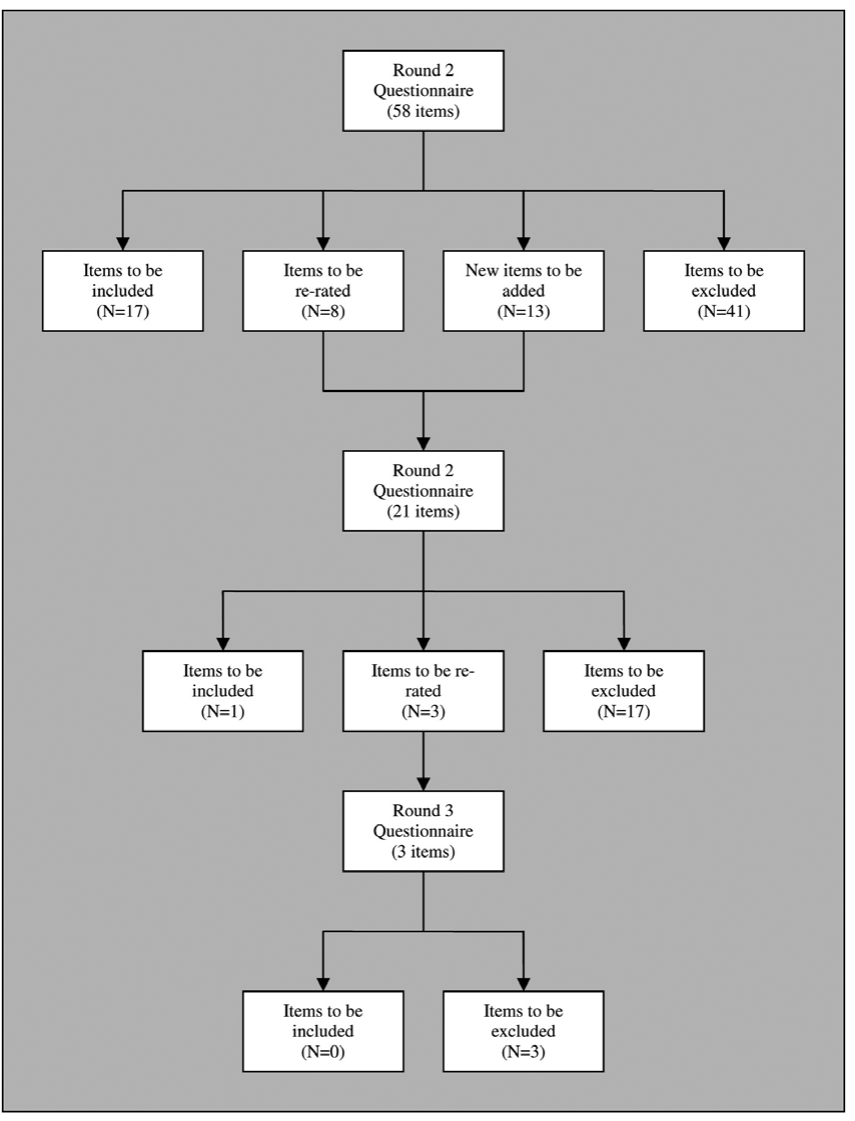
The number of items that were included, excluded and re-rated in the 3 consensus rounds of the study.

Items rated as *essential *or *important *by 80% or more of the consumer and professional panels were accepted for inclusion in the guidelines. If they were endorsed by 80% or more of one panel but not the other panel, or by 70–80% of both panels, they were re-rated in the subsequent round. Items which met neither condition were rejected. Before the second and third rounds of the study, each participant was sent a summary of the results of the previous round, listing which items had been accepted, which had been rejected, and which were to be re-rated. When an item was to be re-rated by the panellists, they were provided with their own response and a table outlining how many people in each group had endorsed the item. They were told that they did not have to change their responses when re-rating an item, but that if they wished to, they would have the opportunity to do so. For a summary of the progress of the items in the three rounds, please see Figure 1.

The suggestions made by the panel members in the first round were reviewed by the working group and used to construct new items for the second round. Suggestions were accepted if they represented a truly new idea, could be interpreted unambiguously by the working group, and were actions. Suggestions were rejected if they were near-duplicates of items in the questionnaire, if they were too specific, too general, or were more appropriate to therapy than first aid. The following examples show how we decided to include or exclude items from the questionnaire;

"*I take my daughter to the swimming pool so that she can enjoy the feeling of her body working and doesn't despise it all.*"

This item was considered too specific to include because it focussed on a specific activity enjoyed by a person engaging in NSSI (swimming) and a specific motivation for NSSI (despising her body).

"*Just be there in the present.*"

This item is very general and difficult to interpret. It does not distinguish between being physically close to the person or available generally to help.

"*Help to discern the function of the behaviour and develop new strategies for fulfilling the same function; if person needs to feel pain, hold ice or exercise hard; if person is dissociating, reconnect with the body by pampering and self-soothing.*"

This is a suggestion which is more appropriate to therapy.

In addition, the wording of some items was criticised by panel members. When this occurred, the items were re-written and included in the next round. For example, "The first aider should only seek professional mental health care if the person asks them to" was re-written removing the word 'only' to reflect that there are other situations where seeking professional help might be important.

## Results

Table 2 shows the continuity of participation across the three rounds. By the third round, no carers remained engaged with the process.

**Table 2:** Study participation at each round

	Pre-Delphi questionnaire	Round 1	Round 2	Round 3
Consumers	16	13	13	9
Carers	3	2	0	0
Professionals	26	21	17	16

Table 3 shows the rates of acceptance, rejection, and re-rating of the items in each round of the questionnaire. Of the 66 items included in the first round, 17 were accepted, 41 were rejected, and 8 met criteria for re-rating. An additional 13 new items were created from suggestions made by the panellists. Of the 21 items included in the questionnaire for the second round, 1 was accepted, 17 were rejected, and 3 met criteria for re-rating. In the second round, there was no option to suggest new items for the third round. Of the 3 items included in the questionnaire for the third round, none were accepted. Of the total of 79 statements rated by the panels, 18 were accepted (See Table 4 for a categorised list of accepted items).

**Table 3:** Items accepted, rejected and re-rated at each round

	Number of items	Items to be included	Items to be re-rated	New items to be added	Items to be excluded
Round 1	66	17	8	13	41
Round 2	21	1	3	n/a	17
Round 3	3	0	n/a	n/a	3

**Table 4:** Statements accepted as mental health first aid guidelines

**Item:**	**Round:**
**Section 1: If the first aider has interrupted someone who is in the process of injuring themselves....**	
... they should express their concern.	1
... they should ask whether they can do anything to alleviate the distress.	1
... they should remain calm, and avoid expressions of shock or anger.	1
... they should ask whether any medical attention is needed.	1
... they should intervene in a supportive and non-judgemental way.	1
If the person has harmed themselves by taking an overdose of medication or consuming poison, the first aider should call an ambulance as the risk of permanent harm or death is high.	1
**Section 2: If the first aider suspects someone has been injuring themselves**	
The first aider should try to avoid a strong negative reaction to the self-injury and discuss it calmly with the person.	1
The first aider should not ignore the injuries, instead acknowledging to the person that they have noticed them.	1
The first aider should not ask the person about self-injury until they have reflected on their own state of mind and are sure they are prepared to calmly deal with the answer.	1
The first aider should understand that self-injury is a coping mechanism.	1
The first aider should avoid taking a punitive stance such as threatening the withdrawal of care.	1
The first aider shouldn't trivialise the feeling or situations which have led to the self-injury.	2
**Section 3: Avoiding self-injury**	
The first aider should keep in mind that 'stopping self-injury' should not be the focus, but look at ways to relieve the distress.	1
The first aider should encourage the person to speak to someone they trust next time they feel the urge to self-injury.	1
**Section 4: Harm minimisation**	
The first aider should ensure that adequate first aid supplies are accessible to the person.	1
**Section 5: Professional help**	
The first aider should encourage the person to seek professional help.	1
The first aider should only seek professional mental health care if the self-injurious behaviour is having an impact on the person's normal functioning (such as the ability to attend school or go to work).	1
The first aider should call an ambulance regardless of the person's wishes if the injury is life-threatening, such as arterial bleeding.	1

The guidelines were developed from these accepted statements and sent to all panellists for comment. Only feedback related to readability and structure was incorporated. The guidelines are appended to this article (see Additional file 1).

## Discussion

The aim of this project was to find statements which were broadly acceptable to professionals and consumers, and to develop first aid guidelines for NSSI from these statements. We have achieved this. However, the items which were not included in the guidelines, because they were acceptable to only one group, highlight some important differences in priorities between people who self-injure and the professionals who may treat them. Two major differences were brought to light, concerning the need of the consumers to be understood and accepted, and the professionals' priorities regarding emergency medical help.

### Consumers' priorities

A number of items related to respect and the right to make choices were endorsed by the consumers. Every consumer endorsed an item which read "the first aider should not ask the person about NSSI until they have reflected on their own state of mind and are prepared to calmly deal with the answer." In the first round, less than two-thirds of the professionals agreed, perhaps reflecting a belief that the priority should be to take action rather than wait for a better moment; however, the item was ultimately accepted. Conversely, all but one consumer agreed that the first aider should respect the person's right to injure themselves, a view endorsed by only half of the professionals. A less dramatic difference is that while 83% of consumers felt it was important to let the person talk about the feelings motivating the NSSI, only 59% of the professionals agreed.

### Professionals' priorities

Professionals agreed that emergency help should be sought in situations where it is suspected that the person has broken a bone, has injured an eye, or is suicidal. They also endorsed seeking professional help if the self-injurious behaviour is escalating over time. These items were not strongly endorsed by the consumers, though they were not summarily rejected either. Most of the professionals (82%) also endorsed doing something pleasant (such as having a hot bath or listening to music) rather than acting on the urge to self-injure, but only 58% of the consumers agreed. One consumer, in a written comment, said that being encouraged to do something nice instead of injuring herself always made her feel that no-one understood how bad she felt, likening it to being given paracetamol for a brain tumour.

### Carer recruitment

It is not known why, when the rates of NSSI are so high, we had so much difficulty in recruiting carers. However, NSSI is often very secretive behaviour and many people go to great lengths to hide their injuries. It is possible that many people are unaware that the person they are caring for is engaging in NSSI.

### Differences between consumers' views

In both the initial questionnaire, and in comments added to the first Delphi round, as well as in correspondence with panellists, some very significant differences were apparent between consumers. It may be that these differences explain the low rate of item acceptance. About half of the consumers suggested and endorsed items which reflected how NSSI has become a part of their life, for example, carrying a kit at all times which contained both first aid items such as dressings and antiseptic, and items they used to injure themselves such as razor blades. They also said that they liked it if someone offered to dress their wounds and did not feel that stopping their self-injurious behaviour was a priority for them. The other half felt that carrying a kit would make it too easy to start seeing NSSI as a part of themselves and would encourage self-identification as 'a cutter'. This group also felt that, while 'stopping NSSI' was not a goal in itself, they hoped that with therapy and support there would come a time in the future when NSSI was no longer needed in their lives. This group's responses were often more aligned to the professionals' views.

### Writing the Guidelines

It was important to the research team to avoid making the guidelines read like a list of 'dos' and 'don'ts'. Therefore, the accepted items were incorporated into a plain language document.

Because there are so many different terms used to describe NSSI, and many of these are also used to described suicide attempts and other behaviours, a paragraph was added to the beginning of the document explaining what we mean by 'NSSI'. In this paragraph, we also indicate that when the injury is actually a suicide attempt or the person is also suicidal, these guidelines are not appropriate. The guidelines for suicidal thoughts and behaviours are referenced so that first aiders can give the most appropriate help. One item which was accepted by the panels said that emergency medical help needed to be sought if the injury was life-threatening, such as a cut resulting in arterial bleeding, so we added information which would allow a first aider to determine whether bleeding was arterial.

### Limitations

One limitation of this study is the small number of panel members, particularly in the carers' panel. Indeed, the carers contributed items for inclusion in the questionnaires, but their responses were excluded from analysis when only one panellist remained. It is important as well to reiterate that all panellists were recruited from developed, English-speaking countries so the guidelines may not be generalisable to other countries or to minority cultures within those countries. Furthermore, these guidelines cannot stand alone, as they do not address the underlying psychological distress or mental illness which leads an individual to injure themselves. These guidelines need to be used in conjunction with the others in this series, including first aid for depression, first aid for psychosis, and first aid for suicidal thoughts and behaviours [40-42]. Guidelines can be downloaded from Mental Health First Aid Australia [49].

The guidelines we have developed may not be appropriate for use by all people in all situations. Future work will need to focus on the roles and responsibilities of different professional groups and situations, and the feasibility of first aid actions for the different groups. For example, correctional officers, teachers and other school staff may all need separate guidelines, tailored to their responsibilities, abilities and professional boundaries.

A final point is that the opportunity to offer first aid for self-injurious behaviours will only present itself if the person, deliberately or accidentally, makes the behaviour known to a first aider. As NSSI is often very secretive behaviour, this may mean that first aiders, especially when they are carers, may need to be aware of some of the more subtle indications that the behaviour is occurring. Future guidelines may need to include a section on these more indirect signs.

## Conclusion

This process has proven that it is possible to develop first aid guidelines which are acceptable both to professionals and to people who have engaged in NSSI in the past. The next priority is to develop strategies for evaluating the use of the guidelines. Where the guidelines are used as the basis for first aid training, efforts need to be made to evaluate their impact on the first aiders' helping behaviours and on the recipients of the first aid, as far as this is possible. This will assist researchers to develop an evidence base for mental health first aid initiatives. It is our hope that appropriate first aid, especially when the behaviour is not yet entrenched, may improve outcomes for individuals engaging in NSSI.

We encourage readers to distribute these guidelines to interested community and carers' groups. Many people who have struggled with NSSI wear scars which remind them daily of the pain which precipitated the behaviour, and it is our hope that in the future, with the continued efforts of the clinical and research communities, fewer people will have such scars to regret.

## Competing interests

The authors declare that they have no competing interests.

## Authors' contributions

CMK and AFJ prepared the manuscript. All authors reviewed the manuscript. AFJ and BAK developed the methodology. CMK did the literature search and wrote the first draft of the questionnaire. All authors contributed to the development of later versions of the questionnaire. CMK wrote the attached guidelines. All authors reviewed and suggested improvements to the guidelines.

## Pre-publication history

The pre-publication history for this paper can be accessed here:



## Supplementary Material

Additional file 1 First aid guidelines for deliberate non-suicidal self-injury. This file may be distributed freely, with the authorship and copyright details intact. Please do not alter the text or remove the authorship and copyright details. Click here for file
