# Supplementary material for: Development of mental health first aid guidelines for deliberate non-suicidal self-injury: A Delphi study
Source: BMC Psychiatry. 2008 Jul 23;8:62. doi: 10.1186/1471-244X-8-62 (PMC2518920; doi:10.1186/1471-244X-8-62)
Supplement: Additional file 1 — First aid guidelines for deliberate non-suicidal self-injury. This file may be distributed freely, with the authorship and copyright details intact. Please do not alter the text or remove the authorship and copyright details. [file 1471-244X-8-62-S1.pdf]

MENTAL HEALTH FIRST AID

DELIBERATE SELF-INJURY

FIRST AID GUIDELINES

Purpose of these Guidelines

These guidelines are designed to help members of the public to provide first aid to someone who is deliberately injuring themselves, but is not suicidal. The role of the first aider is to assist the person until appropriate professional help is received or the crisis resolves.

Development of these Guidelines

The following guidelines are based on the expert opinions of a panel of mental health consumers, carers and clinicians from Australia, New Zealand, the UK, the USA and Canada about how to help someone who may be deliberately injuring themselves. Details of the methodology can be found in: (…)

Although these guidelines are copyright, they can be freely reproduced for non-profit purposes provided the source is acknowledged.

Enquiries should be sent to: Professor Tony Jorm, ORYGEN Research Centre, Locked Bag 10, Parkville, VIC 3052, Australia,
email: ajorm@unimelb.edu.au.

How to use these Guidelines

These guidelines are a general set of recommendations about how you can help someone who may be deliberately injuring themselves. Each individual is unique and it is important to tailor your support to that person's needs. These recommendations therefore may not be appropriate for every person who is deliberately injuring themselves. Also, the guidelines are designed to be suitable for providing first aid in developed English-speaking countries. They may not be suitable for other cultural groups or for countries with different health systems.

An important note:

Self-injury can indicate a number of different things. Someone who is hurting themselves may be at risk of suicide. Others engage in a pattern of self-injury over weeks, months or years and are not necessarily suicidal. These guidelines are to assist you if the person you are helping is not suicidal, but is injuring themselves for other reasons. If the person you are assisting is suicidal, please refer to the guidelines entitled *First aid for suicidal thoughts and behaviours.*

What is deliberate self-injury?

There is a great deal of debate about what self-injury is and how it is different to suicidal behaviour. Many terms are used to describe self-injury including *self-harm, self-mutilation, cutting and parasuicide.*

It is not easy to tell the difference between self-injury and a suicide attempt. Some people argue that anyone who injures themselves must be suicidal even if they are not conscious of it. Others say that it is the person's intentions which count. The only way to know is to ask the person directly if they are suicidal. In this document we are discussing only self-injury which is not intended to result in death. If you are helping someone who is suicidal, please refer to the guidelines entitled *First aid for suicidal thoughts and behaviours.*

This document includes advice on when you should seek professional medical help for a person who has severely injured themselves and is at risk of more serious harm or accidental death, regardless of intent. Remember that accidental death can occur.

There are many different types of self injury. These include:

- Cutting, scratching, or pinching skin, enough to cause bleeding or a mark which remains on the skin
- Banging or punching objects or self to the point of bruising or bleeding
- Ripping and tearing skin
- Carving words or patterns into skin
- Interfering with the healing of wounds
- Burning skin with cigarettes, matches or hot water
- Compulsively pulling out large amounts of hair
- Deliberately overdosing on medications when this is NOT meant as a suicide attempt

Adapted from Whitlock et al (2006). Self-injurious behaviours in a college population. Pediatrics, 117:1939-1948.

People who engage in self-injury do so for many reasons. These include:

- To escape from unbearable anguish
- To change the behaviour of others
- To escape from a situation
- To show desperation to others
- To 'get back at' other people or make them feel guilty
- To gain relief of tension
- To seek help
- To die*

* These guidelines are not suitable for helping someone who wishes to die.
Please see the separate guidelines entitled *First aid for suicidal thoughts and behaviours.*

Adapted from Hawton & James (2005). Suicide and deliberate self harm in young people. British Medical Journal, 330:891-894.

3857_07 A4VERSION ORYGENKJ

How should I talk with someone who is deliberately injuring themselves?

If you suspect that someone you care about is deliberately injuring themselves, you need to discuss it with them. If you have noticed suspicious injuries on the person's body, do not ignore them. Instead let the person know that you have noticed the injuries. Avoid expressing a strong negative reaction to the self-injury and discuss it calmly with the person. It is important that you have reflected on your own state of mind and are sure you are prepared to calmly deal with their answer when asking the person about their self-injury.

Understand that self-injury is a coping mechanism, and therefore, 'stopping self-injury' should not be the focus of the conversation. Instead, look at ways to relieve the distress. Do not trivialise the feelings or situations which have led to the self-injury. Do not punish the person, especially by threatening to withdraw care.

What should I do if I witness someone deliberately injuring themselves?

If you have interrupted someone in the act of deliberate self-injury, intervene in a supportive and non-judgmental way. Remain calm and avoid expressions of shock or anger. Express your concern for the person's wellbeing. Ask whether you can do anything to alleviate the distress. Ask if any medical attention is needed.

What about professional help?

Medical emergency

If the person has harmed themselves by taking an overdose of medication or consuming poison, call an ambulance as the risk of permanent harm or death is high. Deliberate overdose is more frequently intended as a suicide attempt, but is sometimes a form of self-injury. Regardless of the person's intentions, emergency medical help must be sought.

If the injury is life-threatening, emergency medical help must be sought. The emergency services should always be called if the person is confused, disoriented or unconscious, or if they have bleeding that is rapid or pulsing.

Obtaining mental health care

You should encourage the person to seek professional help. Self-injury is not an illness in itself, but is usually a symptom of either a mental illness or serious psychological distress which needs treatment. Ensure that the person knows where professional mental health care is available, but do not force them to use it if they are unwilling.

Further information about encouraging a person to seek professional treatment can be found in the other guidelines in this series.

How can I keep the person safe?

Encourage the person to speak to someone they trust the next time they feel the urge to injure themselves. Also, ensure that adequate first aid supplies are accessible to the person.

The MHFA Training & Research Program
ORYGEN Research Centre
Department of Psychiatry
The University of Melbourne AUSTRALIA

Professor Tony Jorm, ORYGEN Research Centre
Locked Bag 10, Parkville VIC 3052 Australia
email: ajorm@unimelb.edu.au

All MHFA guidelines can be downloaded from **www.mhfa.com.au**
